# Human T lymphotropic virus type 1 (HTLV-1) proviral load induces activation of T-lymphocytes in asymptomatic carriers

**DOI:** 10.1186/1471-2334-14-453

**Published:** 2014-08-22

**Authors:** Raimundo Coutinho, Maria Fernanda Rios Grassi, Ana Beatriz Korngold, Viviana Nilla Olavarria, Bernardo Galvão-Castro, Rita Elizabeth Mascarenhas

**Affiliations:** Advanced Laboratory of Public Health/CPQGM – Oswaldo Cruz Foundation (FIOCRUZ), Rua Waldemar Falcão, no 121, Candeal, Salvador, Bahia CEP 40296-710 Brazil; Bahiana School of Medicine and Public Health, Salvador, Bahia Brazil

**Keywords:** HTLV-1, Proviral load, Asymptomatic, Activation

## Abstract

**Background:**

High HTLV-1 proviral load (PVL) is mainly found in infected individuals with HTLV-1-associated myelopathy/tropical spastic paraparesis (HAM/TSP). However one third of asymptomatic carriers may have high PVL.

This study aimed to evaluate the impact of PVL in the activation of T lymphocytes of asymptomatic individuals infected with HTLV-1.

**Methods:**

Membrane activation markers (CD25^+^, CD28^+^, CD45RO^+^, CD69^+^, CD62L^+^, HLA-DR^+^), FoxP3^+^ and intracellular IFN-γ expression were evaluated on both CD4^+^ and CD8+ T-lymphocytes from asymptomatic carriers with PVL ≥ and < 1% of infected cells, using flow cytometry. HTLV-1 proviral load was determined using real-time PCR.

**Results:**

Asymptomatic carriers with PVL ≥ 1% presented a higher frequency of CD4^+^CD25^+^CD45RO^+^ (13.2% vs. 4%, p = 0.02), CD4^+^HLA-DR^+^ (18% vs. 8.3%, p = 0.01) and CD4^+^IFN-γ^+^ (4.5%; 1%, p = 0.01) T-cells, than healthy donors. HTLV-1 PVL was directly correlated with the proportion of CD4^+^CD25^+^CD45RO^+^ T-cells (R = 0.7, p = 0.003). Moreover, a significant increase in the proportion of CD4 + FoxP3+ T-cells was observed in HTLV-1-infected individuals, compared to healthy donors.

**Conclusion:**

HTLV-1 PVL is associated with activation of both CD4^+^ and CD8^+^ T-lymphocytes in asymptomatic individuals. Prospective studies should be conducted to evaluate whether asymptomatic individuals with higher PVL and high immune activation are more prone to developing HTLV-1-associated diseases.

**Electronic supplementary material:**

The online version of this article (doi:10.1186/1471-2334-14-453) contains supplementary material, which is available to authorized users.

## Background

The human T-lymphotropic virus type 1 (HTLV-1) was the first retrovirus known to cause disease in humans, initially isolated from the lymphocytes of a patient with cutaneous T-cell lymphoma in 1980 [1]. An estimated 10 to 20 million individuals are currently infected with HTLV-1 worldwide, mostly clustered in Japan, the Caribbean, Africa, and Latin America, with Brazil having the highest number of infected individuals [2]. This virus is recognized as the etiologic agent of HTLV-Associated Myelopathy/Tropical Spastic Paraparesis (HAM/TSP) [3, 4] and adult T-cell leukemia/lymphoma (ATL) [5], which affect less than 5% of all infected individuals, and occasionally causes HTLV-associated uveitis (HAU) [6] and infective dermatitis in children [7]. Other infectious diseases, such as strongyloidiasis [8] and tuberculosis [9, 10], have also been described at a higher prevalence in patients infected with HTLV-1.

The association with these infectious diseases suggests that HTLV-1 may impair the host immune response, possibly leading to immunosuppression. Similar to the human immunodeficiency virus (HIV), HTLV-1 also integrates its genome into host cells, thereby establishing a persistent chronic infection. While HIV induces a potent immunodepression by destroying CD4+ T-cells, HTLV-1 promotes the spontaneous proliferation of CD4+ and CD8 + T-cell subsets, as well as NK cells [11–13].

HTLV-1-infected individuals frequently present immunological abnormalities, such as increased inflammatory cytokine production and T-lymphocyte activation, as well as a reduced lymphoproliferative response to recall antigens *in vitro*[13, 14]. Moreover, immune system activation occurs more frequently and with greater intensity in individuals with HAM/TSP [14, 15]. These individuals often present alterations in regulatory T-cells [16, 17].

In the HIV infection, the plasmatic viral load is positively correlated with the intensity of T-cell activation, as well as the destruction of CD4^+^ T-lymphocytes and a progression to AIDS [18]. The general immune activation and exhaustion of immune system caused by HIV play also an important role in the immunodepression observed in AIDS patients [19]. By contrast, HTLV-1 proviral load (PVL) is not widely recognized as a biomarker to predict HTLV-associated disease evolution. HTLV-1 PVL is considered low if the proportion of infected PBMCs is lower than 1%, and high if greater than 5% [20]. High PVLs are commonly found in individuals with HAM/TSP [21, 22], infective dermatitis [23] and Keratoconjunctivitis sicca [24]. However, in some cases, asymptomatic individuals may have high PVLs and exhibit an exacerbated inflammatory response [14, 22]. To investigate the association between PVL and immune system activation, the present study evaluated the phenotypic profile of CD4^+^ and CD8^+^ T-cells in asymptomatic individuals infected with HTLV-1.

## Methods

### Subjects

Fifty seven asymptomatic HTLV-1-infected subjects were sequentially selected from the Bahiana School of Medicine and Public Health HTLV reference center (Salvador, Bahia, Brazil). They were included if evaluated neurologic examination was normal and had no clinical complaints. Exclusion criteria were co-infection with HIV and/or HCV, as well as other HTLV-1-associated diseases, such as infective dermatitis, uveitis, ATL. Eleven laboratory staff volunteered as non-infected controls. All samples were screened for HTLV-1/2 antibodies by an enzyme-linked immunosorbent assay (ELISA) (Ab-Capture ELISA Test System – Ortho-Clinical Diagnostics, Inc., Raritan, New Jersey), and positive results were confirmed by Western Blot (HTLV Blot 2.4, Genelabs Technologies, Singapore). The number of evaluated in the immunological assays was as follow: spontaneous proliferation n = 28 HTLV-1-infected individuals, activation markers on CD4 and CD8^+^ T-subsets n = 10 and CD4^+^FoxP3^+^ n = 15 HTLV-1-infected individuals and 5 uninfected controls). This study was approved by the Ethical Committee of the Oswaldo Cruz Foundation (FIOCRUZ). Informed written consent was obtained from all enrolled individuals and the FIOCRUZ Institutional Review Board approved this study.

### Proliferation assay

Blood samples were collected from all study subjects in heparin tubes and peripheral blood mononuclear cells (PBMC) were isolated using a Ficoll-Paque Plus density gradient centrifuge (GE Healthcare Bio-Sciences AB, Uppsala, Sweden). Cultures of 10^5^ PBMCs were incubated in triplicate for five days (37°C, 5% CO_2_) in RPMI-1640 medium (Sigma Chemical Co., St. Louis, MO, USA) supplemented with 2 mM L-glutamine, 1 mM nonessential amino acids, 1 mM sodium pyruvate, 100 U/ml penicillin, 100 g/ml streptomycin and pooled human AB serum (10%) (Sigma). On the last day of incubation, cells were pulsed overnight with 1 μCi [3H]thymidine (specific activity, 2 Ci/mmol; ICN, Costa Mesa, CA). Incorporated [3H]thymidine was measured in terms of mean counts per minute (CPM) using β-radiation counter (MATRIX 9600 direct beta counter; Packard). Spontaneous proliferation of PBMCs was considered when a mean CPM value ≥ 500 due in triplicates (i.e., three times the mean counts per minute measured in uninfected control cells) was obtained.

### Flow cytometry

50 μl of whole blood was incubated for 15 min at room temperature with anti-CD4 (BD Pharmingen Technical) and the following monoclonal antibodies: CD45RO, CD25, CD69, CD62L, CD28, and HLA-DR (Immunothec, a Beckman Coulter Company). Erythrocytes were subsequently lysed with FACS™ lysing solution (Becton-Dickinson Immunocytometry System, San Jose, CA, USA). After a final wash, the cells were fixed in PBS containing 1% paraformaldehyde. Alternatively, for detection of intracellular FoxP3, 80 μl of whole blood was incubated for 15 min at room temperature with anti-CD3 and CD4 monoclonal antibodies (Becton Dickinson, Mountain View, Calif.), after 15 min the cells were washed twice with PBS-BSA-Saponin 0.2% and blocked with AB serum 1% for 5 min. After that, the cells were incubated with anti-FoxP3 for 30 min. The cells were washed and fixed. Analyses were performed using FACSaria II (Becton Dickinson, Mountain View, Calif.) and the software FlowJo 7.5 (Tree stars, San Diego). At least 10^5^ events were analyzed per sample.

### Detection of intracellular IFN-γ

PBMCs (2 × 10^5^ cells/well) were cultured in RPMI 1640 (Sigma Chemical Co., St. Louis, MO) medium, supplemented with 25 mM of HEPES, 2 mM of L-glutamine, 1 mM nonessential amino acids, 1 mM sodium pyruvate, 100 U/mL of penicillin, 100 g/mL of streptomycin and 10% pooled human AB serum (Sigma), and plated in triplicate in 96-well U-bottom plates (Costar, Cambridge, MA), then incubated for 4 h at 37°C under 5% CO_2_. Next, monensin and brefeldin (3 μg/ml) in AB serum (10%) (Sigma) were added to each culture, and then reincubated for 16 h. The cells were then washed with PBS-BSA-PFA-azide (2 mL) and stained with monoclonal anti-CD4, and antiCD3 antibodies for 15 min at 4°C. The cells were then fixed in PBS containing 4% paraformaldehyde 200 μl for 20 min at room temperature, followed by two washing cycles with 0.1% PBS-BSA-Saponin. Positive control cells were cultured with 2 μg/ml phytohaemagglutinin (PHA) (Sigma). To conduct intracellular cytokine staining, the cells were then reincubated for 30 min with anti-IFN-γ-PE monoclonal antibodies or isotype controls, then washed with 0.1% PBS-BSA-Saponin, and washed again with PBS-BSA-PFA-azide. Cells were analyzed using a FACSort flow cytometer and data was interpreted by Cellquest software (Becton Dickinson, Mountain View, Calif.).

### Proviral load

PBMCs were obtained from EDTA blood using density gradient centrifugation and cryopreserved until use. DNA was extracted using a DNA extraction system (Qiagen, Hilden, Germany). HTLV-1 proviral load was quantified using a real-time TaqMan polymerase chain reaction (PCR) method, as previously described [25]. Briefly, SK110/SK111 primers were used to amplify a 186 bp fragment of the *pol* gene and dual TaqMan probe (50-FAM/50 VIC and 30-TAMRA) was attached at 4,829–4,858 bp of the HTLV- 1 reference sequence (HTLVATK). Albumin DNA was used as an endogenous reference and HTLV-1 PVL was calculated as the ratio of [(HTLV-1 DNA average copy number)/(albumin DNA average copy number)] × 2 × 10^6^ and expressed as the number of HTLV-1 copies per 10^6^ PBMCs.

### Statistical analyses

Data are expressed as median and interquartile range (25th percentile and the 75th percentile). Kruskal–Wallis non-parametric analysis of variance with the Bonferroni-Dunn multiple comparison tests was used to compare healthy donors, asymptomatic with PVL ≥ 1% and < 1% of infected cells groups. The Fisher exact chi-square test was used to compare lymphoproliferation frequencies. The correlations were performed by Spearman correlation test. Significant differences were considered for p <0.05. GraphPad Prism 5 (La Jolla, CA) Software was used for all statistical analyses.

## Results

**Figure 1:**
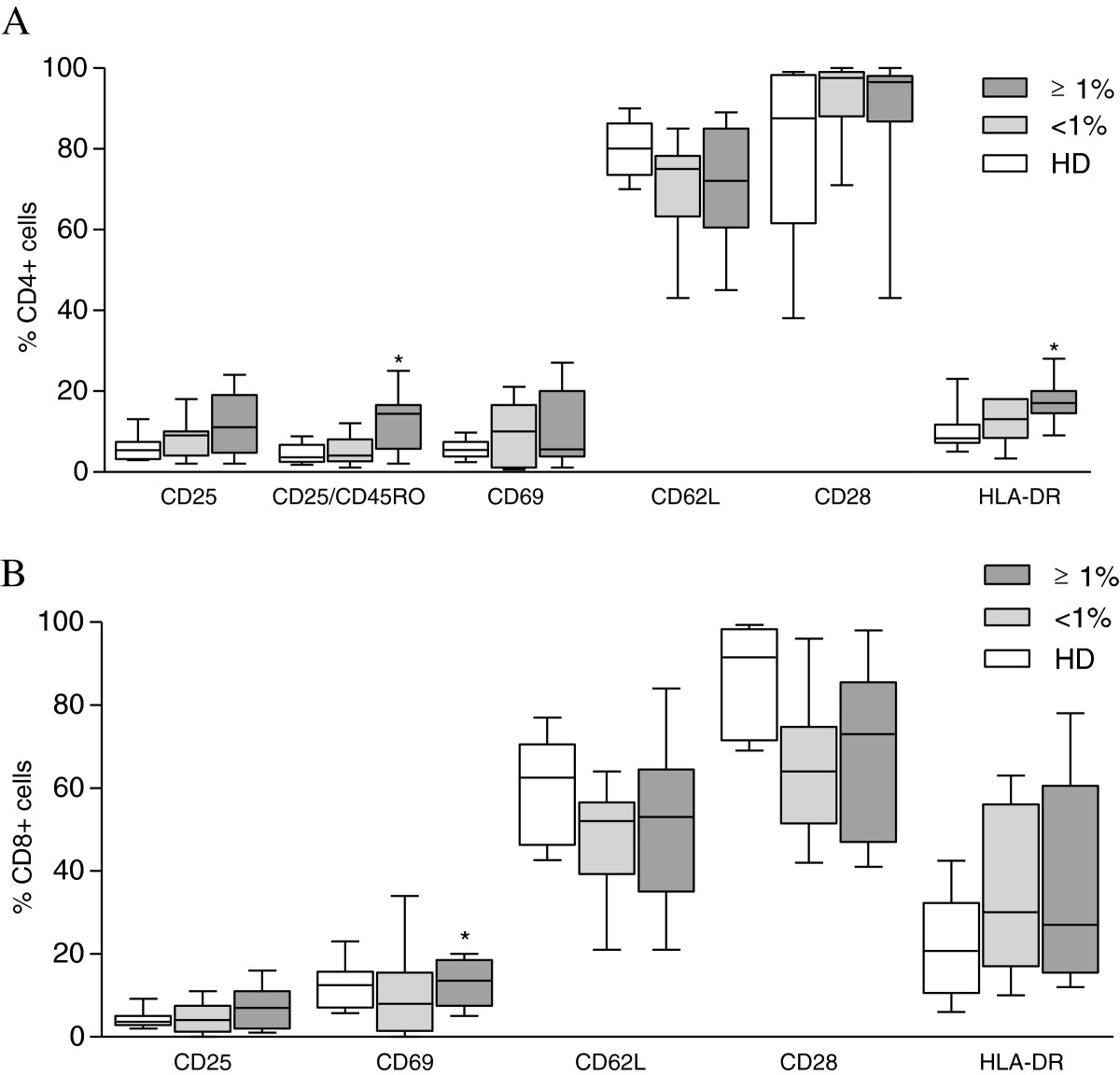
**Activation profile of CD4+ (A) and CD8+ (B) T-lymphocytes from asymptomatic HTLV-1-infected individuals.** Flow cytometry was performed using fresh whole blood samples. Data represents median and interquartile range of 20 asymptomatic HTLV-1infected individuals grouped according to HTLV-1 PVL expressed as ≥1% (10 individuals) and <1% of infected cells (10 individuals) and 10 healthy donors (HD). Kruskal–Wallis test with the Bonferroni-Dunn multiple comparisons. The level of significance was set at P < 0.05.

The median PVL in all HTLV-1-infected carriers was 1.5% of infected cells (IQR, interquartile range 0.12-5.3%), 60% of them had PVL higher than 1% of infected cells. There were no statistically significant differences in markers of cellular activation of CD4^+^ T-lymphocytes between PVL ≥ 1% and <1% HTLV-1-infected groups (Figure 1A). The proportion of both CD4^+^CD25^+^CD45RO^+^ and CD4 + HLA-DR^+^ T-cells from infected individuals with PVL ≥ 1% was higher than healthy donors (13.2% vs 4.0%, p = 0.02; 18.0% vs. 8.3%, p = 0.02, respectively).

Moreover, the frequency of CD4^+^CD25^+^CD45RO^+^ T-cell subset was directly correlated to the HTLV-1 PVL (R = 0.7, p = 0.003).

Concerning the CD8^+^ T-cell subset (Figure 1B), the frequency of CD8^+^CD28^+^ cells was similar between PLV ≥ 1% and <1% groups. A lower frequency of cells expressing CD28 was observed in HTLV-1-infected individuals with PVL <1% (median 64%, IQR 51-75%) compared to healthy donors (median 91%, IQR 71-98%) (p = 0.01).

**Figure 2:**
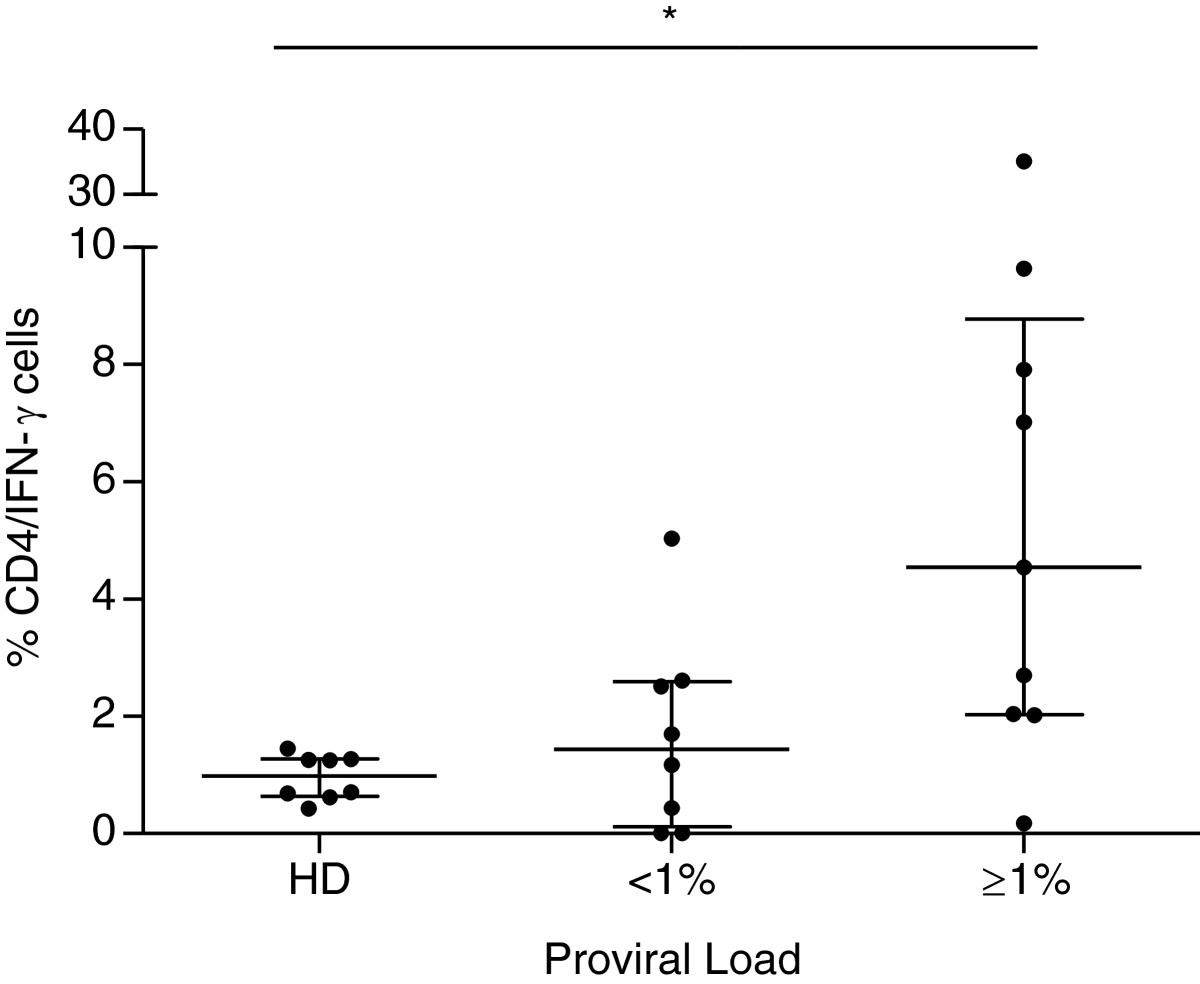
**Intercellular detection of IFN-γ from asymptomatic HTLV-1 infected individuals.** PBMCs (2 × 10^5^ cells/well) were cultured for 18 hours. Data are presented as median (interquartile range). P-value: Kruskal–Wallis test with the Bonferroni-Dunn multiple comparisons.to compare HTLV-1 PVL ≥1% (n = 10) and <1% infected cells (n = 10) groups and healthy donors (HD, n = 10). The level of significance was set at P < 0.05. *p = 0.02.

**Figure 3:**
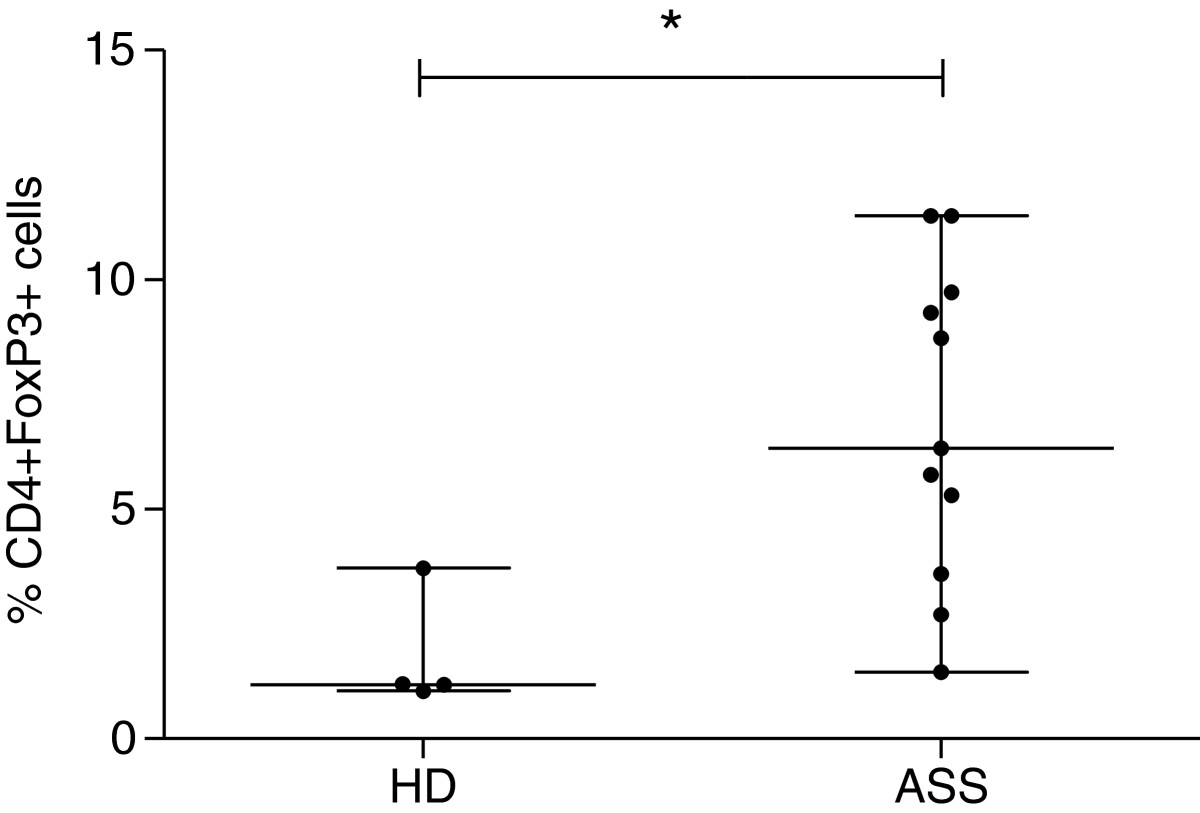
**CD4 + FoxP3+ T-cell frequency in both HTLV-1-infected and healthy donors.** Data are presented as median (interquartile range). The Mann–Whitney U-test was used to compare healthy donors (HD; n = 5) and asymptomatic HTLV-1-infected individuals (ASS n = 15). ASS individuals had PVL < 1% infected cells, except one 26% of infected cells. *p = 0.02.

A higher frequency of CD4^+^IFN-γ^+^ T-cells (4.5%) was observed in the PVL ≥ 1% group, compared to healthy donors (1%) (P = 0.01), while frequencies of CD8^+^IFN-γ^+^ T-cells was similar for HTLV-1 infected groups and healthy donors (Figure 2). The frequencies of individuals with spontaneous lymphocyte proliferation in the group PVL ≥ 1% (72%, 13 out of 18) was similar to that observed in the group PVL <1% (64%, 7 out of 11) (P = 0.69). There was no difference between the magnitudes of proliferation between both groups. However, considering only patients that presented spontaneous proliferation, a positive correlation between PVL and magnitude proliferation was observed (R = 0.7; P = 0.007). The frequency of CD4^+^FoxP3^+^ T-cells was higher among individuals infected with HTLV-1, compared to healthy donors (P = 0.01) (Figure 3).

## Discussion

The present study demonstrated that the HTLV-1 PVL is directly associated with immune system activation in asymptomatic carriers. A higher frequency of activated CD4^+^ T-lymphocytes expressing CD25 + CD45RO+, HLA-DR^+^ and of cells producing IFN-γ were mostly observed in the subgroup of infected individuals with PVL ≥ 1% of infected cells, compared to healthy donors. A cut-off value of 1% for PVL was chosen to distinguish between low and elevated levels in accordance with the literature review by Golçalves et al. [20], which considered <1% as low, between 1-5% as intermediate and >5% as high. Although the median PVL was determined to be 1.5%, a highly variable percentage of infected cells was observed in included individuals (interquartile range of 0.12-5.3%). When the cut-off of 1.5 was selected, no significant differences were detected in the results presented herein. As such, the 1% PVL cut-off value was chosen as a representative value.

Activation of T-lymphocytes has been reported more often in HAM/TSP patients. In those, an expansion in the number of CD4^+^ and CD8^+^ T-lymphocyte subpopulations that present an higher expression of activation molecules such as CD25 and HLA-DR, a decrease in the expression of CD28 costimulatory molecule, compared to asymptomatic carriers [15, 26]. An exacerbated production of proinflammatory cytokines, such as IL-2, TNF-α, IFN-γ, IL-6, and IL-15 have also been reported more frequently in patients with HAM/TSP [14, 27–29]. However, in some asymptomatic HTLV-1-infected individuals the activation of the immune system is similar to those with HAM/TSP [14, 28]. The influence of HTLV-1 PVL on the immune system was not addressed in these studies.

HTLV-1 PVL represents the number or percentage of host cells harboring a viral copy integrated into a host DNA genome. In the last few years, numerous studies have demonstrated a clear association between high PVL and development of HAM/TSP and of other inflammatory conditions such as infective dermatitis and keratoconjunctivitis sicca. Patients with these conditions have a PVL consistently higher than asymptomatic carriers [22, 24, 30]. Recently, our group suggested a cutoff of 5% infected cells as best the value to differentiate HAM/TSP patients from asymptomatic individuals. However, it was observed that about one third of asymptomatic individuals have PVL that exceed this this level [22].

Furthermore, the majority of infected individuals in this study presented spontaneous PBMC lymphoproliferation which was directly correlated with the PVL. Classically, the highest frequency and magnitude of proliferation are found in HAM/TSP [31, 32] although a lower frequency of infected asymptomatic individuals also presents similar levels of proliferation [13, 14].

The memory CD4^+^CD45RO^+^ T-cells are the main target for HTLV-1 infection and are preferentially involved in the spontaneous proliferation. It is estimated that the rate of proliferation of memory T-cells induced by the virus reach 3% per day [33].

The immune response is partially effective maintaining PVL stable over time, probably due to the cytotoxic response mediated by CD8^+^ T-lymphocytes that destroys infected cells. However, the immune response is unable to clear the infection [33–35]. Regarding the subpopulation of CD8^+^ T cells, our study showed a reduced expression of CD28 in the HTLV-1-infected group, although statistically significant only in the subgroup with PVL <1% compared to healthy donors.

Low expression of CD28 on CD8^+^ T-subset has been described primarily in patients with HAM/TSP [36]. Conversely, in other chronic or persistent infections such as schistosomiasis, Chagas’ disease, and HIV infection a decrease of CD28 expression is also found [35, 37, 38].

CD28 is a costimulatory molecule expressed on T lymphocytes that interacts with natural ligands CD80 and CD86 located on antigen-presenting cells that result in cell activation. The reduction on CD28 expression found herein may represent a deactivation pathway of the immune system.

On the other hand, a fivefold increase in the frequency of regulatory T cells (CD4^+^FoxP3^+^) was observed in the group infected with HTLV-1, compared to healthy donors. These results were similar to those obtained by other studies [17, 39] indicating that the virus drives an expansion of regulatory T-cells. This expansion however would not be sufficient to control cell activation induced by the HTLV-1 infection. However, it has been reported that Forkhead box P3 (FOXP3) protein may be transiently expressed on activated T CD4^+^ cells and its expression does not necessarily convey regulatory function [40, 41]. In the present study, we were unable to evaluate the function of these cells.

## Conclusions

In summary, the results presented herein indicate that intermediate HTLV-1 PVL is associated with activation of both CD4^+^ and CD8^+^ T-lymphocytes in asymptomatic individuals. Prospective studies should be conducted to evaluate whether asymptomatic individuals with higher PVL and high immune activation are more prone to developing HTLV-1-associated diseases. The HTLV-1 PVL may be a relevant marker in monitoring asymptomatic individuals.

## Authors’ original submitted files for images

Below are the links to the authors’ original submitted files for images. Authors’ original file for figure 1 Authors’ original file for figure 2 Authors’ original file for figure 3
