# Supplementary figures and images for: Human T lymphotropic virus type 1 (HTLV-1) proviral load induces activation of T-lymphocytes in asymptomatic carriers
Source: BMC Infect Dis. 2014 Aug 22;14:453. doi: 10.1186/1471-2334-14-453 (PMC4148537; doi:10.1186/1471-2334-14-453)

A

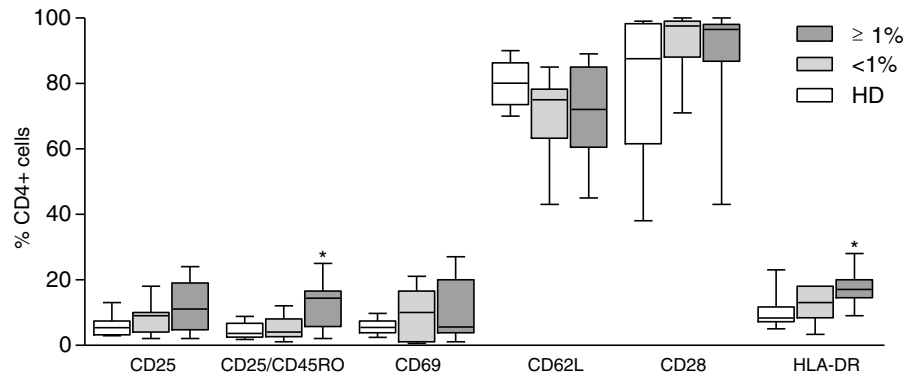

B

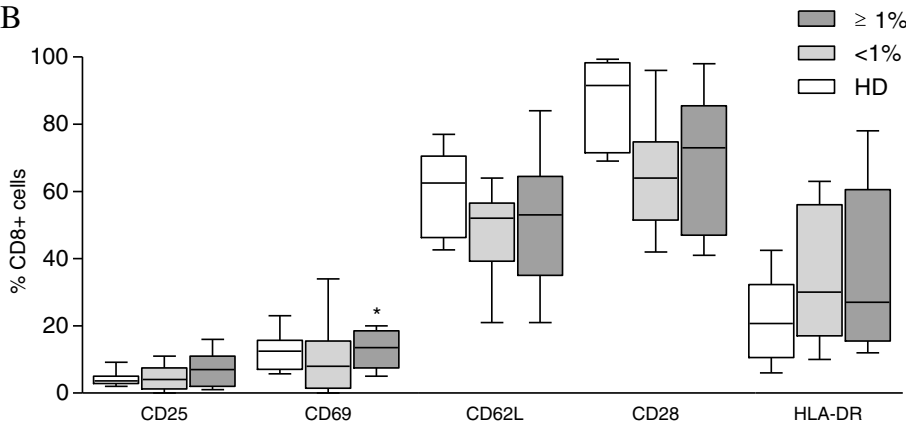

Supplement: Supplementary file 1 — Authors’ original file for figure 1 [file 12879_2014_3747_MOESM1_ESM.pdf]

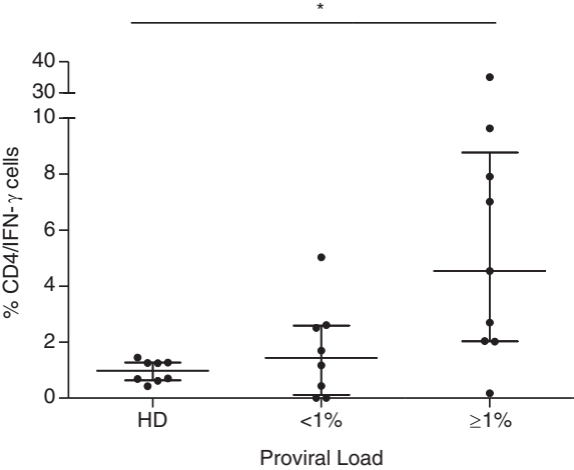

Supplement: Supplementary file 2 — Authors’ original file for figure 2 [file 12879_2014_3747_MOESM2_ESM.pdf]

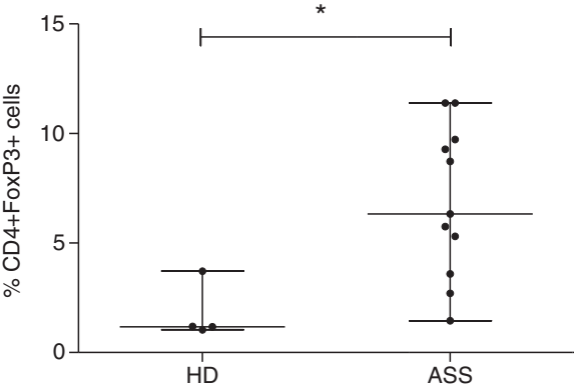

Supplement: Supplementary file 3 — Authors’ original file for figure 3 [file 12879_2014_3747_MOESM3_ESM.pdf]
